# Evaluation of the Bio-Rad Geenius HIV-1/2 test as a confirmatory assay

**DOI:** 10.1186/1471-2334-14-S2-P71

**Published:** 2014-05-23

**Authors:** I Montesinos, J Eykmans, ML Delforge

**Affiliations:** 1 Erasme Hospital, Brussels, Belgium

## Introduction

HIV testing algorithms include a supplemental assay to confirm and differentiate HIV-1 and HIV-2 on repeated reactive samples by a 4th generation EIA screening test (EIA). In this study we evaluate the recently CE-marked Bio-Rad Geenius HIV1/2 (Geenius) confirmatory assay, a single use immunochromatographic test, in comparison with the MP Diagnostic HIV Blot 2.2 (WB).

## Methods

A total of 161 serum samples were tested by Geenius test: 72 nonreactive samples by EIA (Liaison XL Murex and/or VIDAS bioMérieux), 8 indeterminate samples by WB confirmed negative after follow up, 5 low reactive samples by EIA negative by WB and confirmed negative after follow up, 44 HIV-1 reactive samples by EIA and WB, 5 HIV-2 reactive samples, 1 HIV-1/HIV-2 co-infection sample, 15 HIV-1 non-B subtype samples (8 CRF02, 1 CRF01, 1 CRF11, 1 CRF15, 2 subtype G, 1 subtype F1, 1 subtype C) and 11 confirmed HIV-1 early seroconversion samples. The samples were tested according to the manufacturer’s guidelines. Geenius cassettes were read and interpreted by an automated reader utilizing a proprietary algorithm. The diagnostic performance and the quality management of the test were analyzed.

## Results

Overall sensitivity for Geenius assay was 92%. Five of 11 early seroconversion samples were tested positive, 4 negative and 2 indeterminate. The overall sensitivity of WB was 88%. After excluding early seroconversion samples, the sensitivity reaches 100% for both assays. All HIV-1 non-B subtype samples were tested positive. The ability to differentiate HIV-1 and HIV-2 was as follow: 2 out of the 5 HIV-2 reactive samples were tested positive HIV-2, 2 positive HIV-2 with HIV-1 cross reaction, and 1 HIV positive untypable. The co-infection HIV-1/HIV-2 sample was tested HIV positive untypable. The overall specificity for the Geenius assay was 96%. All 5 low reactive samples by EIA, negative by WB were tested negative by Geenius. Two out of the 8 indeterminate samples by WB confirmed negative were tested indeterminate and one invalid, the other 6 were negative. After excluding these last 13 samples, the specificity of Geenius assay reached 100%. In comparison with WB, the Geenius assay is markedly less time consuming (<30 minutes), allows full traceability, automatic reading and interpretation.

## Conclusions

The Bio-Rad Geenius HIV1/2 confirmatory system represents a reliable alternative to other confirmatory assays in HIV testing algorithms and provides clear improvement in quality management.

